# Supplementary material for: Attachment Performance of Stick Insects (Phasmatodea) on Plant Leaves with Different Surface Characteristics
Source: Insects. 2022 Oct 19;13(10):952. doi: 10.3390/insects13100952 (PMC9604322; doi:10.3390/insects13100952)
Supplement: Supplementary file 1 [file insects-13-00952-s001.zip › insects-1977913-supplementary.pdf]

insects

Supplementary Materials:

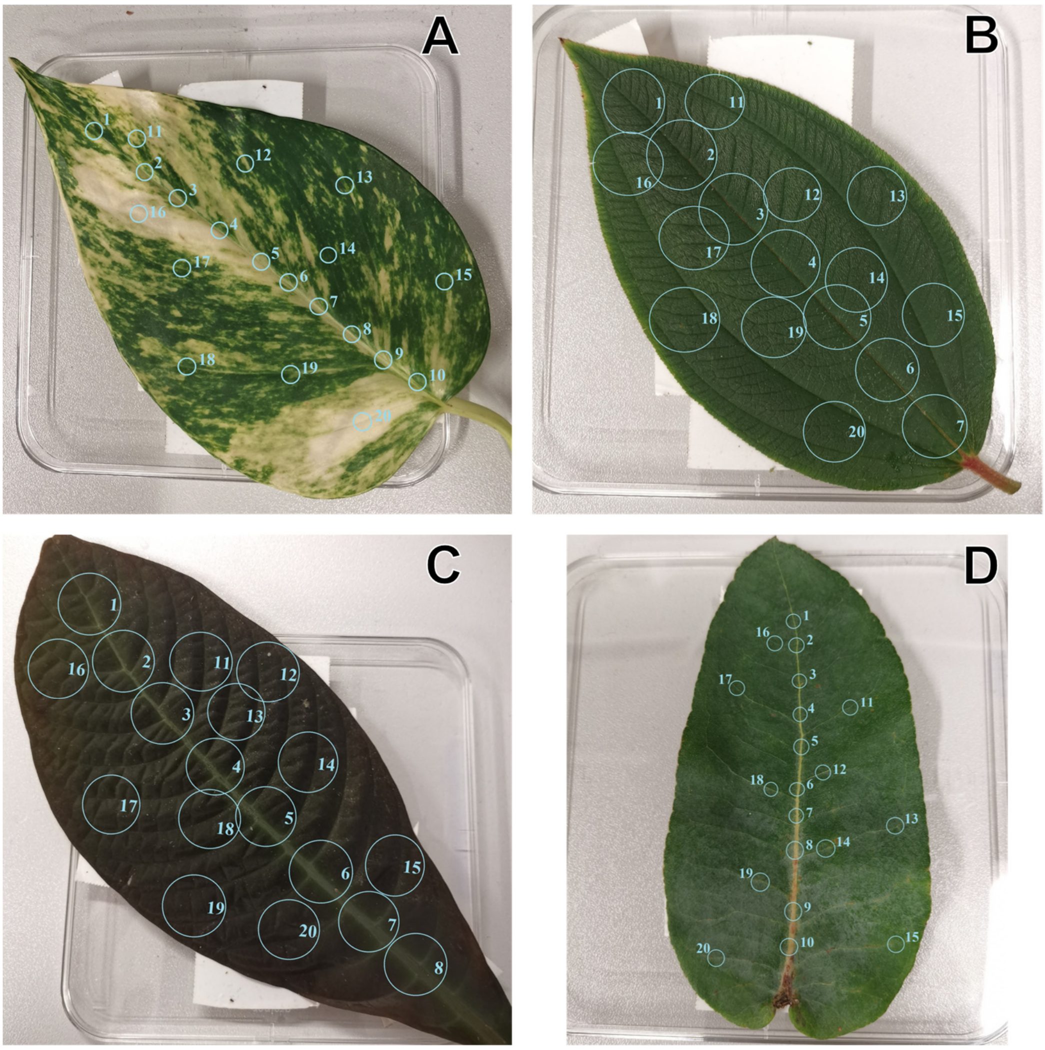

Figure S1. Localisation of the measurements for surface characterization. (**A**) *Ep. aureum*, (**B**) *T. urvilleana*, (**C**) *H. ghiesbreghtii*, (**D**) *Eu. globulus*.

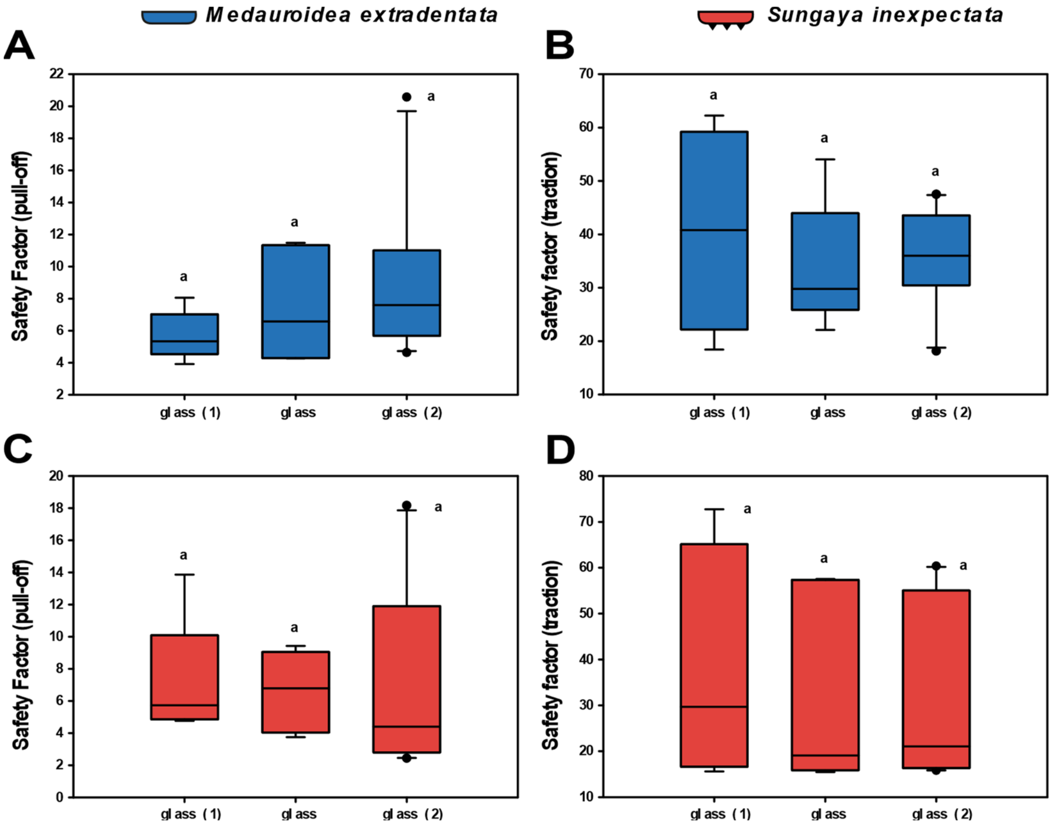

Figure S2. Contamination effect measurement with glass as control substrate. (**A**) Pull-off force measurement with *M. extradentata*. (**B**) Traction force measurement with *M. extradentata*. (**C**) Pull-off force measurement with S. inexpectata. (**D**) Traction force measurement with *S. inexpectata*. Statistically equal groups within a graph are marked with the same letter, tested through Kruskal-Wallis One Way ANOVA on Ranks (d.f. = 2, $N_{glass1,glass} = 5$, $N_{glass2} = 10$; A: H = 2.677, P = 0.262; B: H = 0.643, P = 0.725; C: H = 0.894, P = 0.639; D: H = 0.894, P = 0.832). The boxes indicate 25th and 75th percentiles, whiskers define the 10th and 90th percentiles and the line within the boxes represents the median. Outliers are shown as individual points.

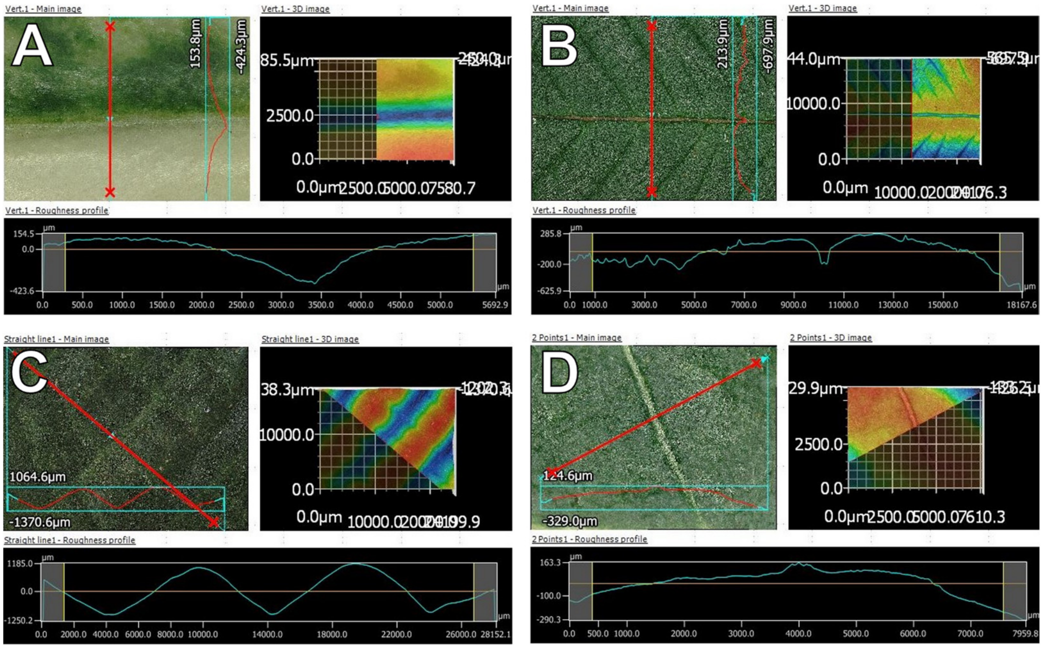

Figure S3. Exemplary 3D scanning images of the adaxial leaf surface with KEYENCE VR-300. In each composite figure the upper left picture is the scanned area, the upper right image is a topographic heat map of the scanned area and below is the roughnes profile. (**A**) *Ep. aureum* area 4, (**B**) *T. urvilleana* area 3, (**C**) *H. ghiesbreghtii* area 17, (**D**) *Eu. globulus* area 12 (see Figure S1).

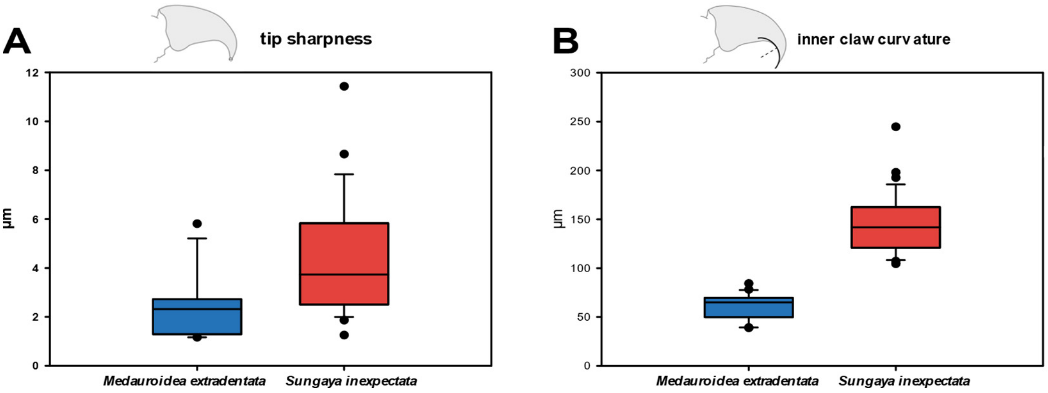

Figure S4. Tip sharpness (**A**) and inner curvature (**B**) of *M. extradentata* and *S. inexpectata*. The groups are all statistically different, tested through Mann-Whitney Rank Sum Test (A: $N_{M.extradentata}$=12, $N_{S.inexpectata}$=25, P=0.003; B: $N_{M.extradentata}$=24, $N_{S.inexpectata}$=35, P>0.001). The boxes indicate 25th and 75th percentiles, whiskers define the 10th and 90th percentiles and the line within the boxes represents the median. Outliers are shown as individual points.
